# Loss of Sodium/Hydrogen Exchanger NHA2 Exacerbates Obesity- and Aging-Induced Glucose Intolerance in Mice

**DOI:** 10.1371/journal.pone.0163568

**Published:** 2016-09-29

**Authors:** Christine Deisl, Manuel Anderegg, Giuseppe Albano, Benjamin P. Lüscher, David Cerny, Rodrigo Soria, Elisa Bouillet, Stefano Rimoldi, Urs Scherrer, Daniel G. Fuster

**Affiliations:** 1 Division of Nephrology, Hypertension and Clinical Pharmacology, Bern University Hospital, University of Bern, Bern, Switzerland; 2 Institute of Biochemistry and Molecular Medicine and Swiss National Centre of Competence in Research (NCCR) TransCure, University of Bern, Bern, Switzerland; 3 Department of Clinical Research, Bern University Hospital, University of Bern, Bern Switzerland; 4 Division of Cardiology, Bern University Hospital, University of Bern, Bern, Switzerland; 5 Facultad de Ciencias, Departamento de Biologia, Universidad de Tarapaca, Arica, Chile; University of Bremen, GERMANY

## Abstract

We previously demonstrated that the sodium/hydrogen exchanger NHA2, also known as NHEDC2 or SLC9B2, is critical for insulin secretion by β–cells. To gain more insights into the role of NHA2 on systemic glucose homeostasis, we studied the impact of loss of NHA2 during the physiological aging process and in the setting of diet-induced obesity. While glucose tolerance was normal at 2 months of age, NHA2 KO mice displayed a significant glucose intolerance at 5 and 12 months of age, respectively. An obesogenic high fat diet further exacerbated the glucose intolerance of NHA2 KO mice. Insulin levels remained similar in NHA2 KO and WT mice during aging and high fat diet, but fasting insulin/glucose ratios were significantly lower in NHA2 KO mice. Peripheral insulin sensitivity, measured by insulin tolerance tests and hyperinsulinemic euglycemic clamps, was unaffected by loss of NHA2 during aging and high fat diet. High fat diet diminished insulin secretion capacity in both WT and NHA2 KO islets and reduced expression of NHA2 in WT islets. In contrast, aging was characterized by a gradual increase of NHA2 expression in islets, paralleled by an increasing difference in insulin secretion between WT and NHA2 KO islets. In summary, our results demonstrate that loss of the sodium/hydrogen exchanger NHA2 exacerbates obesity- and aging-induced glucose intolerance in mice. Furthermore, our data reveal a close link between NHA2 expression and insulin secretion capacity in islets.

## Introduction

Sodium/hydrogen exchangers (NHEs) are ion transport proteins found across all phyla of uni- and multicellular organisms and exchange monovalent cations with protons across lipid bilayers. In mammals, 13 NHE isoforms are currently known [1,2]. NHA2, also known as SLC9B2 or NHEDC2, is a recently cloned, poorly characterized NHE isoform [3]. Previous studies suggested that NHA2 is the correlate of the long sought sodium/lithium countertransporter that was linked to the pathogenesis of diabetes mellitus and essential hypertension in humans [2,4,5].

While NHA2 is ubiquitously expressed on tissue level, it is mainly confined to specialized cells within individual organs, e.g. osteoclasts in the bone or distal tubules of the kidney [4,6,7]. We recently reported that NHA2 is present in human and rodent β-cells of the endocrine pancreas [8]. Islets isolated from NHA2 knock-out (KO) mice displayed an insulin secretion deficit upon stimulation with glucose or the sulfonylurea tolbutamide. Similar findings were obtained when NHA2 was knocked-down by RNA interference in the murine β–cell line Min6 [8].

Confocal microscopy and subcellular fractionation studies revealed that NHA2 localizes to endosomal structures in β–cells, and depletion or loss of NHA2 caused inhibition of clathrin-dependent endocytosis in primary β–cells and Min6 cells [8]. Given the known tight interaction of endo-and exocytosis in β–cells, these results suggested that disrupted endo-exocytosis coupling may be the primary cause for the insulin secretion deficit observed [9,10]. The exact role of NHA2 in β–cell endosomes, however, remains unclear at the moment, but seems not to involve endosomal pH homeostasis [8].

To gain more insights into the role of NHA2 on systemic glucose homeostasis, we studied the impact of NHA2 deficiency during the physiological aging process and in the setting of diet-induced obesity.

## Materials and Methods

### Mice

All animal experiments were in accordance with the Swiss Animal Welfare Law and were approved by the local Veterinary Authority (Veterinary Office of the Kanton Bern). Mice had free access to water and chow and were maintained on a 12 hours light/12 hours dark cycle at room temperature (23°C). Normal diet (F1850; 20.5% protein, 7.2% fat, 61.6% carbohydrate) and high fat diet (F3282; 20.5% protein, 36% fat, 35.7% carbohydrate) were purchased from Bio-Serv, Frenchtown, NJ. Both diets were otherwise identical.

Generation of NHA2 KO mice lacking exon 7 of the *NHA2* gene was described in detail previously [8]. All mice used in this study were males and completely backcrossed into C56BL/6J background (> 10 generations). Completeness of backcrossing was verified by microsatellite marker analysis, as described [8].

### Intraperitoneal glucose (IPGTT) and insulin (IPITT) tolerance tests

Blood glucose and serum insulin concentrations were measured in male mice of indicated age after a 6 to 12 AM 6 hr fast (ip. glucose tolerance test) or at random fed state at 2 PM (ip. insulin tolerance test) as described [8,11,12]. Blood glucose was measured before and after intraperitoneal injections of glucose (2g/kg or 1 g/kg) or insulin (1 U/kg Actrapid HM, Novo Nordisk, Denmark) with a Contour glucose monitor (Bayer Healthcare, Germany) by tail vein sampling at indicated time points in duplicates. The upper detection limit of the glucose monitor used was a glucose concentration of 33.3 mmol/L, values exceeding this limit were counted as 33.3 mmol/l. Serum insulin (CrystalChem, Downers Grove, IL, USA), serum leptin (CrystalChem), serum adiponectin (CrystalChem) and plasma glucagon (Mercodia, Uppsala, Sweden) concentrations were determined by ELISAs.

### Hyperinsulinemic euglycemic clamp studies

Hyperinsulinemic euglycemic clamp studies were performed as described [13]. Clamps were done in freely moving mice after 12 weeks of high fat diet. Three days before the clamp studies, mice were anesthetized with isoflurane, and an indwelling catheter to be used for insulin infusion was inserted into the vena cava through the femoral vein, sealed under the back skin, and exteriorized and glued at the back of the neck. On the day of the clamp, after a 6-hour fast, insulin (18 mU x kg^-1^ x min^-1^, a dose known to suppress hepatic glucose production) was infused into the femoral vein for 2 hours. Throughout the infusion, blood samples (3.5 μL) were collected every 10 minutes from the tip of the tail vein for the determination of the blood glucose concentration, and euglycemia (5.5 mmol/l) was maintained by periodic adjustment of a variable infusion of 15% glucose. The steady state glucose infusion rate was calculated as the mean of the values obtained every 5 minutes during the last 30 minutes of the clamp.

### Isolation of islets and *in vitro* insulin release assays

Pancreata were perfused *in situ* with collagenase solution and islets isolated exactly as described [8,14]. After overnight incubation in RPMI medium with 11 mM glucose, islets were washed twice with KRBH containing 2 mM glucose and then placed in 12-well plates (10 islets/well) containing 1 ml of KRBH with 2 mM glucose and pre-incubated for 2.5 hrs at 37°C. Insulin secretion into the KRBH buffer was measured for 2 hrs. After this time, supernatants were harvested, plates put on ice and total cellular insulin extracted by addition of acid ethanol (70% ETOH, 1.5% HCL conc.). Secreted and cellular insulin (CrystalChem) was determined by ELISA.

### Total liver lipid extraction

Liver tissue (30 mg) was homogenized in 1x PBS and lipids were extracted in a chloroform/methanol (2:1) mixture. Total liver lipids were determined by a sulpho-phospho-vanillin reaction as described previously [15,16].

### RNA isolation and real-time PCR

RNA isolation was performed using Trizol reagent (ThermoFisher Scientific, Waltham, MA, USA) as described [5,8]. Real-time PCR was performed using pre-synthesized Assays-on-Demand (AoD; Life Technologies/ABI, Rotkreuz, Switzerland) on an ABI ViiA 7 System. The following AoDs were employed: NHA2 (Mm01313329_m1), GAPDH (Mm99999915_g1). The amount of NHA2 relative to GAPDH mRNA was calculated using the ΔCt method.

### Statistical Analysis

Statistical significance measurements were performed by Student *t* test or one-way ANOVA for multiple comparisons. All statistical tests were two-sided and a *p* < 0.05 was considered statistically significant. Data are shown as means and error bars indicate SEs.

## Results

### Loss of NHA2 KO worsens aging-induced glucose intolerance in mice

**Fig 1:**
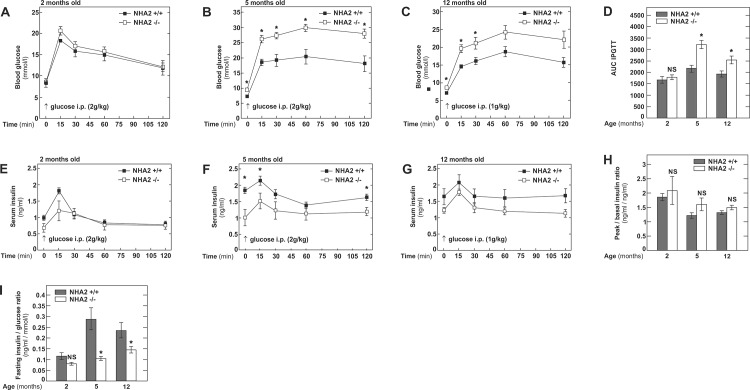
Intraperitoneal glucose tolerance tests in NHA2 WT and KO mice fed normal chow diet at different ages. A) Blood glucose concentrations following ip. glucose challenge (2g/kg body weight) at 2 months; n_+/+_ = 8, n_-/-_ = 8. B) Blood glucose concentrations following ip. glucose challenge (2g/kg body weight) at 5 months; n_+/+_ = 9, n_-/-_ = 9. C) Blood glucose concentrations following ip. glucose challenge (1g/kg body weight) in 12 months old mice; n_+/+_ = 9, n_-/-_ = 9. D) Area under the curve (AUC) of intraperitoneal glucose tolerance tests (IPGTT) at 2, 5 and 12 months of age. E) Serum insulin concentrations following ip. glucose challenge (2g/kg body weight) at 2 months; n_+/+_ = 8, n_-/-_ = 8. F) Serum insulin concentrations following ip. glucose challenge (2g/kg body weight) at 5 months; n_+/+_ = 9, n_-/-_ = 9. G) Serum insulin concentrations following ip. glucose challenge (1g/kg body weight) at 12 months; n_+/+_ = 9, n_-/-_ = 9. H) Peak (15 minutes) to basal (0 minutes) insulin ratios during ip. glucose challenges at 2, 5 and 12 months of age. I) Fasting insulin to glucose ratios at 2, 5 and 12 months of age. Data are mean values ± SEM, (* p<0.05, WT *vs* KO; NS = not significant, WT *vs* KO).

To study the age-dependent impact of *NHA2* gene inactivation on glucose homeostasis, we studied 3 different groups of male mice, aged 2, 5 and 12 months, respectively. All mice were males in a C57BL6/J background and either wild-type (WT) or bearing a targeted mutation in the *NHA2* gene (NHA2 KO) [8]. As shown in Fig 1A, 1D and 1E fasting blood glucose was not different between 2 months old WT and NHA2 KO mice and an intraperitoneal glucose load (IPGTT) of 2 g/kg body weight caused similar glycemic excursions and insulin levels in both groups of mice. In 5 months old mice, however, we observed clear differences between the two groups of mice (Fig 1B and 1D). NHA2 KO mice displayed higher fasting glucose and significantly higher glycemic excursions after i.p. glucose challenge (2 g/kg body weight) compared to WT mice. Serum insulin was significantly lower in the fasting state, 15 and 120 minutes after glucose challenge. Glucose challenge in 12 months old mice was performed with a reduced dose of glucose (1g/kg body weight), because 2g/kg body weight induced blood glucose levels in NHA2 KO mice that were consistently exceeding the measurable range of the glucose meter. Blood glucose was significantly higher in 12 months old NHA2 KO mice compared to WT mice in the fasting state and up to 30 minutes after glucose administration (Fig 1C and 1D). Serum insulin levels, however, were not different in 12 months old NHA2 KO mice compared to WT mice (Fig 1G). Basal versus peak insulin ratios during the IPGTTs were not different between the two groups of mice at all ages (Fig 1H).

**Fig 2:**
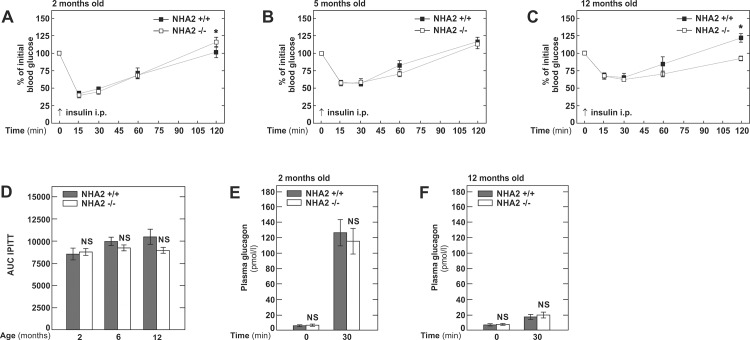
Intraperitoneal insulin tolerance tests in NHA2 WT and KO mice fed normal chow diet at different ages. A) Blood glucose concentration in % of baseline glucose following ip. insulin challenge (1 U/kg body weight) at 2 months; n_+/+_ = 10, n_-/-_ = 10. B) Blood glucose concentration in % of baseline glucose following ip. insulin challenge (1 U/kg body weight) at 5 months; n_+/+_ = 11, n_-/-_ = 12. C) Blood glucose concentration in % of baseline glucose following ip. insulin challenge (1 U/kg body weight) at 12 months; n_+/+_ = 9, n_-/-_ = 12. D) Area under the curve (AUC) of intraperitoneal insulin tolerance tests (IPITT) at 2, 6 and 12 months of age. E) Plasma glucagon concentration at baseline and 30 minutes after ip. administration of insulin (1 U/kg body weight) in 2 months old mice. F) Plasma glucagon concentration at baseline and 30 minutes after ip. administration of insulin (1 U/kg body weight) in 12 months old mice. Data are mean values ± SEM, (* p<0.05, WT *vs* KO; NS = not significant, WT *vs* KO).

Fasting insulin/glucose ratios at 2 months of age were similar in both groups of mice but significantly lower in NHA2 KO mice at 5 and 12 months of age (Fig 1I). Thus, in a next step, we tested the peripheral insulin sensitivity by intraperitoneal insulin tolerance tests (1 U /kg body weight). However, at all ages, sensitivity of NHA2 KO mice to insulin was not different from WT mice (Fig 2A–2D). However, 120 minutes after insulin administration, significant but opposite differences in blood glucose between NHA2 WT and KO mice were observed in 2 and 12 months old mice, respectively (Fig 2A and 2C). Such variations maybe be due to an altered counterregulatory response during hypoglycemia. To exclude that loss of NHA2 affects glucagon secretion by α-cells, we measured plasma glucagon at baseline and 30 minutes after insulin administration. (Fig 2E and 2F). Plasma glucagon was significantly elevated at 30 minutes compared to baseline. The increase was more pronounced in the 2 months old mice, in agreement with the more pronounced hypoglycemia induced by insulin administration. At both time points, however, we detected no differences between NHA2 WT and KO mice, indicating that loss of NHA2 does not alter hypoglycemia-induced glucagon release.

Together, our experiments indicate that loss of NHA2 exacerbates aging-associated glucose intolerance in mice.

### Loss of NHA2 KO worsens obesity-induced glucose intolerance in mice

**Fig 3:**
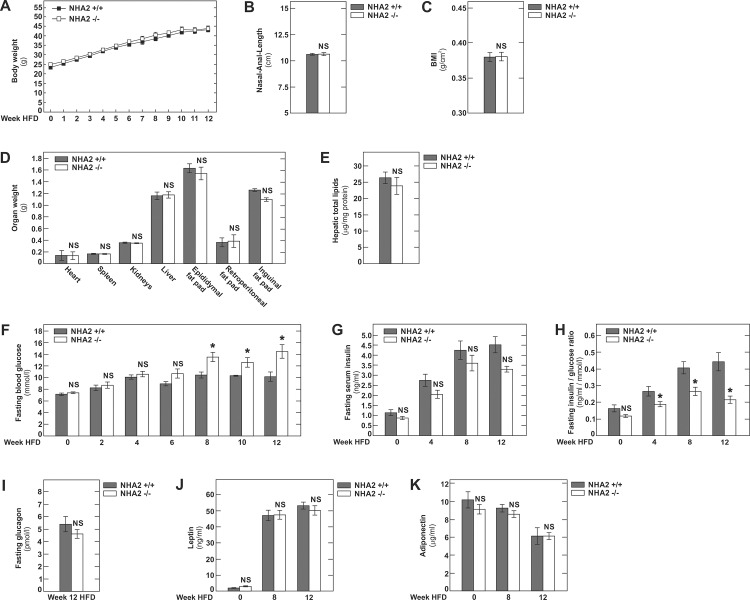
High fat diet in NHA2 WT and KO mice. A) Weight gain of mice during 12 weeks of HFD. HFD was started at 8 weeks of age. B) Nasal to anal length at week 12 of HFD. C) BMI at week 12 of HFD. D) Organ and fat pad weights at week 12 of HFD. E) Hepatic total lipid content at week 12 of HFD. F) Fasting blood glucose concentration at baseline and at indicated week during HFD. G) Fasting serum insulin concentration at baseline and at indicated week during HFD. H) Fasting insulin to glucose ratios at indicated time points during HFD. I) Fasting plasma glucagon concentrations at week 12 of HFD. J) Fasting serum leptin concentrations at baseline and at indicated week of HFD. K) Fasting serum adiponectin concentrations at baseline and at indicated week of HFD. Data are mean values ± SEM, (* p<0.05, WT *vs* KO; NS = not significant, WT *vs* KO).

We next sought to investigate the role of NHA2 during diet-induced obesity. To this end, 8 week old male C57BL6/J WT or NHA2 KO mice were placed on an obesogenic high-fat diet (HFD) for 12 weeks [17]. As shown in Fig 3A, WT and NHA2 KO mice displayed comparable weight gains throughout the HFD. There was also comparable linear growth in both groups of mice and thus no differences were apparent in the body mass index (BMI, g/cm^2^) at the end of the HFD period (Fig 3B and 3C). Similarly, organ weights and total hepatic lipid content of mice sacrificed at 5 months of age after 12 weeks of HFD revealed no differences between WT and NHA2 KO mice (Fig 3D and 3E). In addition, we observed no differences in the size of inguinal, epididymal and retroperitoneal fat pads (Fig 3D). During HFD, fasting blood glucose and fasting insulin levels were measured every two and four weeks, respectively (Fig 3F and 3G). As shown in Fig 3F, fasting blood glucose levels rose continuously with duration of HFD in both groups of mice. Beyond week 8 on HFD, fasting blood glucose was consistently higher in the NHA2 KO mice compared to WT mice (Fig 3F). Fasting serum insulin tended to be lower throughout the HFD period in NHA2 KO mice, but the differences did not reach statistical significance. While the fasting insulin/glucose ratio was similar in both groups of mice at baseline, we found it to be significantly lower in NHA2 KO mice at 4, 8 and 12 weeks of HFD (Fig 3H).

As observed on regular chow, on HFD NHA2 KO mice displayed a fasting plasma glucagon that was not different from WT mice on HFD (Fig 3I).

Given the widespread tissue distribution of NHA2, we next tested if, in addition to insulin secretion, loss of NHA2 affects secretion of other peptide hormones involved in glucose homeostasis. For this, we quantified concentrations of the two major adipokines leptin and adiponectin at baseline and after 8 and 12 weeks of HFD. As shown in Fig 3J and 3K, our results reveal the well known differential secretion pattern of the two hormones by adipose tissue during HFD [18,19]. However, as is the case for glucagon, leptin and adiponectin secretion were unaffected by loss of NHA2.

Together these experiments reveal that ectopic lipid deposition, hepatic steatosis and fat mass gain are similar in NHA2 WT and KO mice during diet-induced obesity. However, lack of NHA2 causes progressive increase of fasting glycemia with a drop of fasting insulin/glucose ratios during obesogenic HFD.

**Fig 4:**
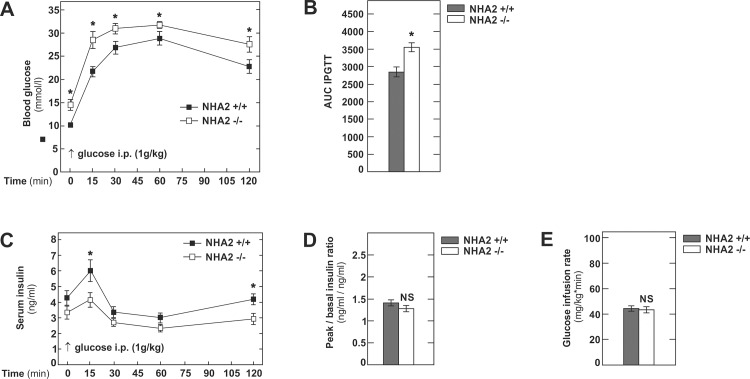
Intraperitoneal glucose tolerance test and hyperinsulinemice euglycemic clamp in NHA2 WT and KO mice after 12 weeks of HFD. A) Blood glucose concentrations following ip. glucose challenge (1g/kg body weight); n_+/+_ = 12, n_-/-_ = 10. B) Area under the curve (AUC) of the intraperitoneal glucose tolerance test (IPGTT). C) Serum insulin concentrations following ip. glucose challenge (1g/kg body weight); n_+/+_ = 12, n_-/-_ = 10. D) Peak (15 minutes) to basal (0 minutes) insulin ratios after 12 weeks of HFD. E) Glucose infusion rates during hyperinsulinemic euglycemic clamps; n_+/+_ = 8, n_-/-_ = 8. Data are mean values ± SEM, (* p<0.05, WT *vs* KO; NS = not significant, WT *vs* KO).

We next tested the functional significance of NHA2 inactivation for glucose disposal by performing intraperitoneal glucose challenges (1g/kg body weight) to WT and NHA2 KO mice on HFD. As depicted in Fig 4A and 4B, WT mice were clearly glucose intolerant after 12 weeks of HFD, as reported previously [17]. Mice lacking NHA2 had higher fasting glycemia and displayed significantly higher glycemic excursion during the glucose challenge than WT mice. Serum insulin levels were lower in NHA2 KO mice at 15 and 120 minutes of the IPGTT (Fig 4C). Peak to basal insulin ratio after 12 weeks of HFD was similar in both groups of mice (Fig 4D).

To evaluate peripheral insulin sensitivity, we next performed hyperinsulinemic euglycemic clamps on WT and NHA2 KO mice after 12 weeks of HFD. As evidence by the low steady-state glucose infusion rate, WT mice were insulin resistant after 12 weeks of HFD (Fig 4E). However, the degree of insulin resistance was identical in NHA2 KO mice, indicating that also in the setting of diet-induced obesity, loss of NHA2 per se does not alter insulin sensitivity.

### Age- and HFD-dependent changes in WT and NHA2 KO islets

**Fig 5:**
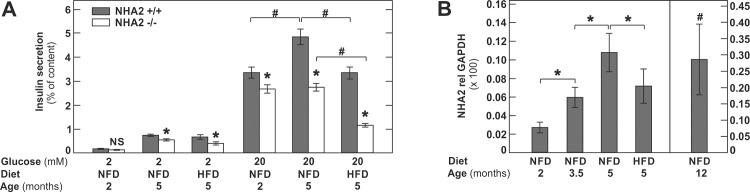
*In vitro* insulin secretion of NHA2 WT and KO islets. A) Basal (2 mM) and glucose (20 mM) induced insulin secretion of islets isolated from 2 or 5 months old mice fed a normal chow (NFD) or from 5 months old mice fed a high fat diet (HFD) for 12 weeks; n = 300 islets per genotype and condition. Data are mean values ± SEM, (* p<0.05, WT *vs* KO; NS = not significant, WT *vs* KO; # p<0.05, WT *vs* WT or KO *vs* KO. B) NHA2 mRNA expression in islets of 2, 3.5, 5 and 12 months old mice fed a normal chow (NFD) or from 5 months old mice fed a high fat diet (HFD) for 12 weeks; n = 300–500 islets of 3–5 mice/group. Data are mean values ± SEM, (* p<0.05, *vs* indicated condition; # p<0.05, *vs* all other conditions).

Together, the phenotype observed in aging mice and in mice with diet-induced obesity suggests that lack of NHA2 causes glucose intolerance, at least partially, due to inappropriate insulin secretion. Interestingly, however, the phenotype of young 2 months old NHA2 KO mice was almost indistinguishable from WT mice, indicating that either well-functioning compensatory mechanisms are in place or that NHA2 expression is very low in islets at this age. To explore this in more detail, we next investigated *in vitro* glucose-induced insulin secretion by islets isolated from 2 and 5 months old mice and from 5 months old mice subjected to a 12 weeks HFD prior to islet isolation. Insulin secretion was quantified in % of total islet insulin content. As shown in Fig 5A, islets isolated from 2 months old NHA2 KO mice exhibited a basal insulin secretion (2 mM glucose) that was not different from WT islets, but displayed a ~20% reduction of insulin secretion compared to WT islets in high glucose medium (20 mM glucose). In contrast, islets isolated from 5 months old mice displayed ~20% reduced basal insulin secretion and ~ 45% reduction of insulin secretion in high glucose. The impact of loss of NHA2 in islets of 5 months old mice fed a HFD for 12 weeks prior to islet isolation was even more pronounced. Basal and high glucose-induced insulin secretion were reduced by ~ 40% and ~60%, respectively (Fig 5A). Furthermore, in islets isolated from normal chow fed mice, we observed an age-dependent increase of insulin secretion, which was blunted in NHA2 KO islets (Fig 5A). Compared to islets isolated from mice fed a normal chow diet, we observed a significant reduction of insulin secretion in both WT and NHA2 KO islets isolated of HFD fed mice (Fig 5A).

Given the age- and diet-dependent differences in insulin secretion between WT and NHA2 KO islets, we next sought to determine the impact of aging and HFD on NHA2 expression in murine WT islets. As shown in Fig 5B, we observed a continuous, age-dependent increase of NHA2 expression in islets. In contrast, HFD caused a significant reduction of NHA2 expression in islets (Fig 5B).

## Discussion

In the present study we evaluated the impact of NHA2 deficiency on glucose homeostasis in mice during physiological aging and diet-induced obesity. Mice used for the study were on a C57BL6/J background, and HFD-induced changes in WT mice were similar as reported previously [17,20,21]. Our results indicate that the impact of NHA2 deficiency on glucose homeostasis islets is augmented by aging and obesogenic HFD. Worsened glucose tolerance in NHA2 KO mice under both conditions was not due to altered peripheral insulin sensitivity. Furthermore, serum insulin levels were similar in NHA2 KO and WT mice. In contrast, islets isolated from NHA2 KO mice displayed significant reductions of glucose-induced insulin secretion when compared to WT islets *in vitro*. These findings suggest the presence of *in vivo* compensatory mechanisms for the reduced islet insulin secretion capacity, e.g. a decreased hepatic insulin clearance or an expansion of islet cell mass in NHA2 KO mice. In addition, however, despite similar insulin levels, NHA2 KO mice were significantly more glucose intolerant during aging and HFD and displayed significantly decreased fasting insulin to glucose ratios compared to WT mice. These results indicate that likely additional, insulin-independent mechanisms of glucose tolerance are disturbed in NHA2 KO mice under these conditions [22]. Clearly, a β-cell-specific NHA2 KO model, which unfortunately is not available at the moment, would be very helpful in the differentiation of β-cell-dependent and β-cell -independent roles of NHA2 in the phenotype observed.

We previously demonstrated that even partial knock-down of NHA2 in Min6 cells or heterozygous loss of NHA2 in islets reduces insulin secretion and that insulin secretion could be rescued by overexpression of WT, but not functionally dead, human NHA2 [8]. These observations suggested a direct link of NHA2 transport activity with insulin secretion in β–cells. Results obtained in the current study demonstrate that NHA2 expression increases significantly during aging. Furthermore, upregulation of NHA2 expression during aging was paralleled by an increased sensitivity of islets towards loss of NHA2, evidenced by an increased difference of insulin secretion between WT and NHA2 KO islets. These results clearly support the notion of a link between NHA2 expression and insulin secretory capacity in islets. Low levels of NHA2 expression in islets at 2 months may also explain the fact that we observed only subtle differences *in vitro* between islets of WT and NHA2 KO mice. During obesogenic HFD, NHA2 expression but also insulin secretion dropped significantly in WT islets, but the difference in insulin secretion between the two groups of islets remained large. However, NHA2 expression was still significantly higher in HFD islets than in islets of 2 months old mice. The NHA2 expression in HFD islets was comparable to 3.5 months old normal chow fed mice, where we previously found a ~50% reduction of insulin secretion in NHA2 KO islets [8]. HFD-associated toxicity affects islets on multiple levels by a myriad of factors [23]. It is conceivable that chronic alterations in NHA2 KO islets render them especially sensitive to HFD-associated toxicity. Specific pharmacologic inhibition of NHA2 would allow the differentiation of direct functional consequences from adaptive changes in the islets caused by loss of NHA2 and formally test the concept of a direct link between NHA2 function and insulin secretion. Unfortunately, however, such a specific NHA2 inhibitor is not available at the moment. Nevertheless, our results suggest that a better understanding of the molecular mechanisms governing NHA2 expression in islets may offer opportunities for pharmacologic modulation of NHA2 expression and thus insulin secretion in pancreatic β-cells.

Given the wide tissue distribution of NHA2, it was conceivable that loss of NHA2 may also affect secretion of other peptide hormones engaged in glucose homeostasis, thereby contributing to the phenotype observed in NHA2 KO mice. Our results, however, indicate that glucagon and the two major adipokines leptin and adiponectin are not affected by loss of NHA2. Thus, together, our *in vivo* and *in vitro* data indicate that the phenotype of NHA2 KO mice is caused by direct alterations at the level of the β–cell. Due to the lack of a β-cell specific NHA2 KO mouse model, however, we currently cannot completely exclude the possibility that humoral or non-humoral factors outside of the β–cell contribute to the phenotype observed.

Aging and obesity are believed to be the main causes for the increased prevalence of type 2 diabetes in the last decades [24,25]. While the hallmark of type 2 diabetes is resistance to the action of insulin, diminished or inappropriate secretion of insulin is universally present in type 2 diabetics [26]. Defects in insulin secretion occur early in the pathogenesis of type 2 diabetes, and both reduction in β-cell mass and intrinsic β-cell dysfunction contribute to the defective insulin secretion in type 2 diabetes [26,27]. There is compelling evidence that genetic factors affecting insulin secretion contribute to the pathogenesis of type 2 diabetes [28,29]. While the role of NHA2 in human islets has not been studied to date, human islets exhibit robust NHA2 expression and several single-nucleotide polymorphisms (SNPs) in human NHA2 that alter the transport characteristics were recently described [8,30]. The phenotype of individuals with these functionally relevant SNPs in NHA2 has not been studied to date, but these individuals may be at an increased risk to develop glucose intolerance under certain circumstances (obesity, old age).
